# Bases, positions and computations

**DOI:** 10.1098/rstb.2024.0218

**Published:** 2025-10-20

**Authors:** Karine Chemla

**Affiliations:** ^1^ School of Mathematics and Maxwell Institute for Mathematical Sciences, University of Edinburgh, Edinburgh EH9 3FD, Edinburgh, UK; ^2^ SPHERE, CNRS SHS-Université Paris Cité, Paris, Île-de-France, France

**Keywords:** number base, multiplication, division, reciprocal, cuneiform, Chinese

## Abstract

Ancient sources attest to the introduction of two families of numeration systems using place-value notations. In such systems, the base and its powers are always represented using position. The existence of a base of this kind is reflected by the repetitive character of the algorithms drawing on the place-value notations to execute operations—specifically, multiplicative operations. This article argues that these two families of place-value numeration systems are of a fundamentally different nature, and that their respective nature is revealed by the way algorithms executing multiplicative operations rely on the base. The algorithms associated with Old-Babylonian sexagesimal place-value notations brought into play the number-theoretical properties of the base (its divisibility properties) and features of the sequence of digits writing the number that the choice of the base made prominent (like the last digits of its representation). Other types of computation were independent of the value of the base and could be applied to numbers written using any base. The computations’ iterative character is thus different. To compute a reciprocal, scribes in Old-Babylonian schools relied upon the last digits of the sexagesimal expansion of a number to identify regular divisors and multiply by their inverse; the result was yielded factor by factor. By contrast, multiplications and divisions using decimal place-value notation in Chinese sources dealt with numbers digit by digit. The part played by the base, and hence its meaning, differed deeply.

This article is part of the theme issue ‘A solid base for scaling up: the structure of numeration systems’.

## 1. Introduction

This article focuses on numeration systems that are place-valued. To formulate the topic in the terms of this special issue, it deals with numeration systems in which the base and its powers are expressed using positions in a horizontal sequence of numerical signs, oriented from left to right in descending powers of the base. We use a system of this kind today, when, reading an inscription like ‘123’, we associate ‘2’ with ‘twice ten’ and ‘1’ with ‘one hundred’ in relation to their positions in the sequence of numerical signs. Note that I use the term ‘digit’ to refer to the limited number of numerical signs that occur in these positions. In the numeration system used today, the numerical signs 1, 2, 3 do not have any inner structuring. As will become clear in this present text, this is not the case for the two numeration systems upon which this article concentrates.

Current historiographies of mathematics claim that ancient sources attest to the introduction of two systems that use a place-value principle. The earliest evidence that we have for the first is found in cuneiform sources. Indeed, Old-Babylonian tablets produced in the context of scribal schools at the beginning of the second millennium BCE bear witness to the use of a sexagesimal place-value notation.^1^ Secondly, Sanskrit sources attest to the use of a decimal place-value numeration system—the *Āryabhatīya*, which is believed to have been completed in 499, being the oldest known Sanskrit work that describes a notation of this kind [7, pp. 10−12]. Subsequent Arabic, Syriac and Latin sources testify to the later adoption of the latter notation in the lands around the Mediterranean and then in Europe [8, p. 237]. From the first century onwards, in fact, Chinese sources also reflect the use of a numeration system of this kind—we shall return to this point later.

If we stay at this level in the description of the notation, one may be tempted to draw the conclusion that the two aforementioned types of numeration system are similar. This might lead us to wonder whether they were not historically connected. Such questions have indeed been raised in the historiography. This, for instance, is one of the arguments on the basis of which van der Waerden [9,10] puts forward the idea that a ‘neolithic civilization,’ speaking ‘Indo-European languages’, developed a mathematics that was the common source of mathematics in the Near East and in China [10, p. 46]. For Otto Neugebauer, by contrast, it was clear that the decimal place-value system attested by Sanskrit sources was derived historically from a change of the base sixty into a base ten. This is what he asserts without any argument in Neugebauer [11, vol. 3, p. 1113].

In this article, I argue that the two types of place-value notation attested to—the one in Old-Babylonian sources, and the other, in Arabic, Chinese, Latin and Sanskrit sources—are in fact fundamentally different.^2^ They do not merely differ because the base of the former is sixty and that of the latter is ten. I argue that the ways in which arithmetical operations draw on the base to get the result—that is, the ways in which the operations bring into play the sequences of positions representing the numbers upon which one operates—show that the part played by the base in these two cases is completely different.^3^ In other words, the execution of operations highlights that the base of a place-value numeration system can assume wholly distinct meanings—if we agree to assume that ‘meaning’ derives from use.

To explain this point, I will examine the evidence we have, first in Chinese sources, and then in cuneiform sources, about how multiplicative operations (that is, multiplication and division, as well as root extractions) were carried out. I focus on these operations for a key reason: whether we look at cuneiform sources or at the earliest extant Chinese and Sanskrit sources attesting to place-value numeration systems, they contain procedures to execute operations with place-value notations only for multiplicative operations. The execution of addition and subtraction with the same type of numeration systems appeared only later.^4^ This seems to indicate that place-value numeration systems—that is, numeration systems in which the base number and its powers were written using positions—have emerged together with algorithms carrying out multiplicative operations, rather than being introduced first, and only later being used to execute arithmetical operations.

## 2. The testimony of early China sources

**Figure 1.**
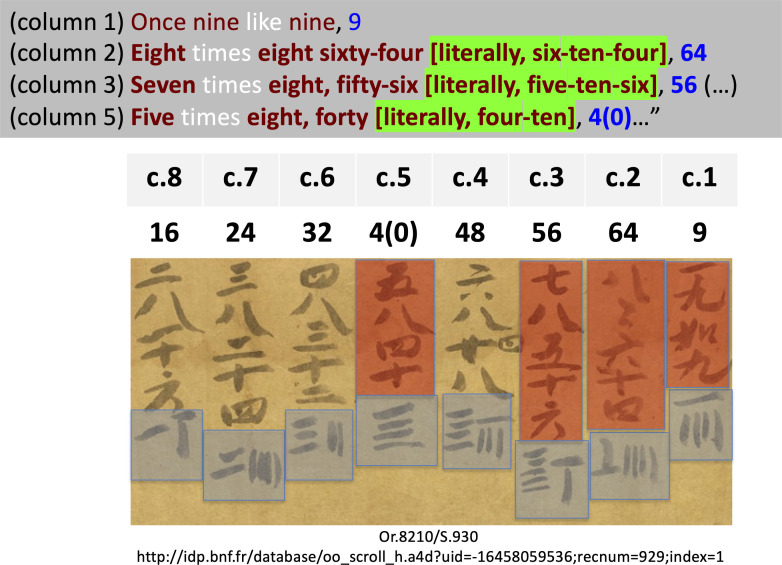
The manuscript partly reproduced here dates from the tenth century and is one of the earliest extant graphic pieces of evidence for the numerical signs that were made with calculating rods since at least the first century CE. The section shown contains part of a multiplication table. The clauses are written in Chinese characters. I have highlighted this part in red, and, for four clauses, I have translated these characters using number words. At the end of each clause, the result is written a second time using ‘rod-numerals’. I have highlighted all these numerical signs in blue, and I have translated them into Arabic numerals. The related Arabic numerals also feature on the top of each column, corresponding to the rod numerals that appear at the bottom. For ease of reference, I have added column numbers, from right to left. From the British Library Collection: Stein Dunhuang scroll Or.8210/S.930 (https://idp.bl.uk/collection/9f11129ec672469eab429509d632c702/). I thank the British Library for granting the permission to use this image.

The earliest extant discursive piece of evidence in a Chinese source for the use of a decimal place-value numeration system is found in *Mathematical Canon by Master Sun* (*Sunzi suanjing* 孫子算經)—I explain below why I use ‘discursive’ here. Qian Baocong [14] dates this work to the year 400, while acknowledging that the received editions bear traces of changes from the eighth century.^5^ The work describes a way of representing numbers with calculating rods, i.e. with material objects. It was not until the tenth century that the available sources include illustrations of representations of numbers with numerical signs that are not Chinese characters (see figure 1), and these illustrations fit the description given in *Mathematical Canon by Master Sun* about the use of material rods. To understand the nature of the numeration system for the period before these illustrations appeared—a system that was only represented on an ephemeral surface with calculating rods—historians must weave together two types of evidence. First, as mentioned above, there are the descriptions such as those given in *Mathematical Canon by Master Sun*. Secondly, ancient mathematical sources describe algorithms for executing multiplicative operations that give us indirect clues about the nature of the underlying numeration systems. I refer to the former type of evidence (the description of numeration systems) as ‘discursive’ evidence, and I use the term ‘clues’ to refer to the latter type, that is, the evidence we can glean indirectly from the algorithms executing operations. Whether we consider Chinese, Sanskrit or Arabic sources, in fact, the nature of our evidence about decimal place-value systems more or less up to the tenth century is of the same two types. The aforementioned work titled *Āryabhatīya*, for example, describes a use of position to write numbers, apparently also on an ephemeral surface, and we need to examine the clues given by algorithms executing multiplicative operations to understand more fully the nature of the numeration system described. As in the case of the Chinese sources mentioned above, neither the text of the description, nor the texts referring to such algorithms contain illustrations of what happened on the ephemeral surface. It remains to be determined precisely at what point such illustrations started to emerge. The nature of the evidence makes the establishment of facts about this type of numeration system quite delicate from a historical viewpoint.

As was just said, for the case of Chinese sources, *Mathematical Canon by Master Sun* gives us the earliest extant discursive evidence for the decimal place-value system. However, the earliest extant clues we have about this numeration system come from an earlier work, *The Nine Chapters on Mathematical Procedures* (*Jiu zhang suanshu* 九章算術), which I date to the first century CE.

In what follows, we will examine in turn the description of the numeration system with ‘rod-numerals’ given in *Mathematical Canon by Master Sun*, and then the texts of an algorithm executing a multiplicative operation with calculating rods, our aim being to observe what these different types of evidence tell us about the use of the base.

The description of the numeration system is rather terse. Let us quote it before offering an interpretation that will be illustrated using figure 1. It reads as follows:

For any method using calculating rods, one first determines the corresponding positions (i.e., the positions involved in the computation as well as the decimal positions corresponding to the decimal parts of the quantities on which one operates—the next sentences dealing with the orientation according to which one puts the rods in the decimal positions). The ones (i.e., units) [correspond to] vertical [rods], the tens to horizontal ones, the hundreds to erected ones, the thousands to reclining ones, [rods corresponding to] the thousands and the tens observing each other (*xiang wang*) (i.e., have the same orientation), [rods corresponding to] the tens of thousands and the hundreds being in conformity with/conforming with each other (*xiang dang*). For six, you do not accumulate (the calculating rods), whereas for five, you [do] not [use a rod] alone.

凡算之法，先識其位，一從十横，百立千僵，千十相望，萬百相當。六不積，五不隻。^6^

Let us begin the discussion of the interpretation of the text with its second part. There, the description associates decimal orders of magnitude expressed using number words (‘ones’, ‘tens’, ‘hundreds’, ‘thousands’ and ‘tens of thousands’) with numerals written using rods in alternating orientations. Number words are the only numerical signs used in the text, the rods being used only on a material support—the ephemeral surface—distinct from the pages of books. The column 7 in figure 1—which comes from a much later document—illustrates how two horizontal rods and four vertical rods were used to indicate, respectively, ‘20’ and ‘4’. Vertical and horizontal rods alternate for the orders of magnitude that follow each other, representing the digits attached to the even and the odd powers of ten, respectively. We thus see that the way rods are used confer an inner structuring to the writing of the digits.

The last sentence of the description translated above addresses the issue of an order of magnitude for which the associated amount is greater or equal to five. When the amount is equal to five (as in 5 or in 52), five rods are used (their orientation corresponding to the order of magnitude); when the amount, by contrast, is strictly greater than five (as in 9 or in 64), a single rod is used for 5, and rods are added for the additional units. This is illustrated for the number ‘9’ in column 1: the four vertical rods still indicate units larger than 5, and a horizontal rod placed on top indicates 5. The cases of the positions with horizontal rods are illustrated by how ‘64’ is written in column 2: horizontal rods still indicate tens, while a vertical rod placed on top refers to the five additional tens.

So far, we have commented on the way in which rods were used to write an amount corresponding to a given order of magnitude. The first part of the text introduces the term ‘position’ (*wei* 位) in a way that is difficult to interpret with certainty. As we will see in the text of the algorithm that executes multiplications in *Mathematical Canon by Master Sun*, this term is used, first of all, to refer to the three rows used to execute a multiplication (middle, lower and upper rows), and the same holds true for the algorithm for division, which is given immediately after that for multiplication; this use of three positions for these operations will remain constant in the traditions that will follow from the canons. Secondly, the term ‘position’ is also used to refer to the digits of the numbers in what appears to have been a decimal place-value system, such as that shown in figure 1. I have argued, in fact, that *The Nine Chapters on Mathematical Procedures* was the earliest extant mathematical document in Chinese that attests to the use of systems of positions, and the work attests to the use of systems of positions of these two types simultaneously ([17], translated as [18]), as if positions were used to write numbers with rods at the same time as systems of positions were used to execute various types of operations. In the description of the numeration system of *Mathematical Canon by Master Sun*, I therefore suggest that the term ‘position’ may have had these two meanings.

The fact that what is described here is indeed a decimal place-value system is confirmed by the clues on the underlying numeration system contained within the algorithms for multiplication, division and root extraction in *Mathematical Canon by Master Sun* and those for root extractions in *The Nine Chapters on Mathematical Procedures* besides. Given the way in which rods are used to write numbers, one may be tempted to object that the numeration system described is centesimal rather than decimal. In a first demonstration that the observation of executions can contribute to the determination of the meaning of the base, the execution of multiplicative operations will show, however, that this is not the case.

To analyse how this numeration system—and in particular how the base number 10—is put into play in computations, let us turn to the text of the algorithm for multiplication given in *Mathematical Canon by Master Sun*. I will quote the text, sentence by sentence, illustrating the interpretation on which all historians agree on the basis of the example of multiplying 57 by 23.^7^ The text begins as follows:

In any multiplication *cheng* method, one puts (on the calculating surface) the corresponding positions (the digits of the multiplied numbers) twice (in two rows), [the positions or digits] in the upper and lower [rows] observing each other (i.e., having their corresponding positions on top of each other with the same orientation of rods). 凡乗之法,重置其位，上下相觀。

**Figure 2.**
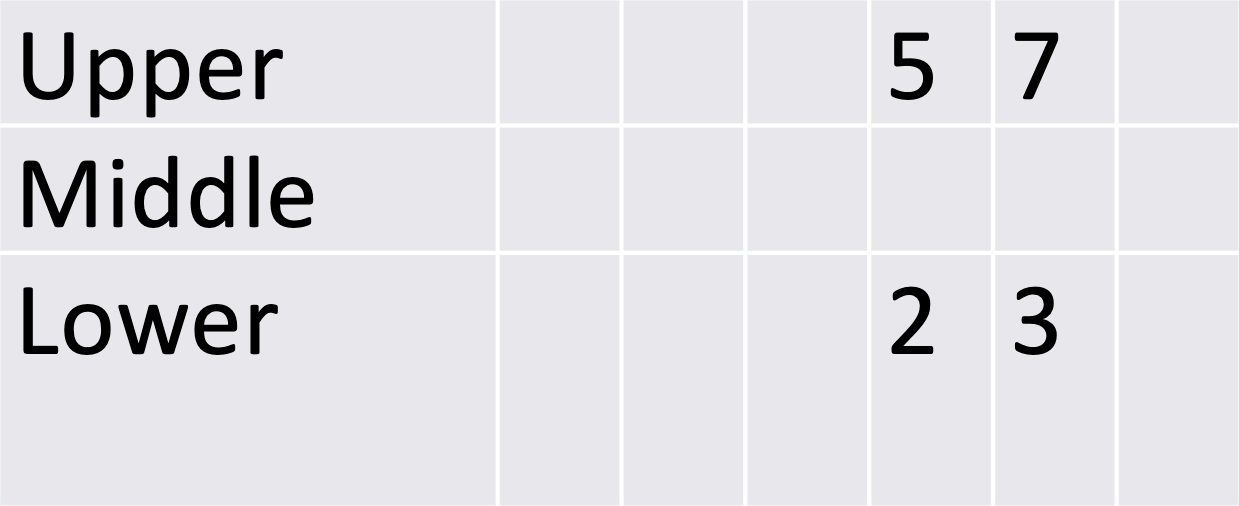
The initial configuration of a multiplication according to *Mathematical Canon by Master Sun.*

As the above sentence—and subsequent part of the text—makes clear, one of the numbers multiplied (i.e. the multiplicand) is set in the upper row, and the other (i.e. the multiplier) is set in the lower row (see figure 2). In the description of the numeration system, rods corresponding to odd powers of ten were said to ‘observe each other’—‘[rods corresponding to] the thousands and the tens observing each other (相望*xiang wang*)’—; this I had interpreted as referring to the corresponding rods having the same orientation. Here, a similar verb (‘observe, watch’ 觀 *guan*) is used to refer to digits placed on top of each other in the initial configuration: digits with the same order of magnitude are written with rods having the same orientation.

The immediately following sentence in the text is essential for our purpose, bringing into relation the order of magnitude of the number represented in the upper row and the motion that must be applied to the representation of the multiplier in the lower row. It reads as follows:

If, in the upper position, there are tens, we make [the number below] move forward (*bu*) to the tens; if there are hundreds, we make [the number below] move forward (*bu*) to the hundreds; if there are thousands, we make [the number below] move forward (*bu*) to the thousands. 上位^8^ 有十步至十，有百步至百，有千步至千。

**Figure 3.**
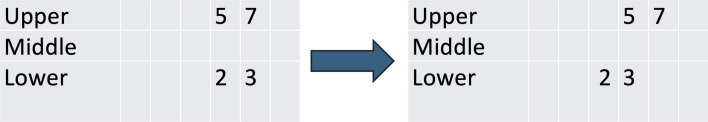
The movement of the multiplier at the beginning of a multiplication according to *Mathematical Canon by Master Sun.*

In our example of 57 multiplied by 23, the multiplier is moved horizontally leftwards so that its digit of the units is in the ‘columns’ or the ‘position of the tens,’ as shown in figure 3. Interestingly, the term ‘column’ (*deng* 等) and the expression ‘position of the tens’ (*shi wei* 十位) both occur in the text of the algorithm for division that follows the one for multiplication. The same type of motion would be applied if the top digit of the multiplicand was in the hundreds or in the thousands. In other words, a movement leftwards applied to the representation of the multiplier corresponds to a multiplication by the order of magnitude of the first digit of the multiplicand. Note that the use of these elementary shifts confirms that the base number is ten—and not a hundred.

Later in the text, in the formulation of the iteration of the first steps, we will see that a horizontal movement of one column rightwards (or ‘backwards’) corresponds to a division by ten. The fact that the algorithm makes use of this motion of the representation of a number to multiply or divide by a power of ten reveals the place-value character of the numeration system in the context of which the operation is executed. What is more, this motion actually characterizes how the positions that write the base and its orders of magnitude are used in the context of this type of execution. Similar shifts are put into play, more broadly, to execute divisions and root extractions in *Mathematical Canon by Master Sun* as well as in *The Nine Chapters on Mathematical Procedures.*^9^ To the best of my knowledge, however, we find no reference to such motions in earlier Chinese mathematical documents, in which specific tables are given for the execution of multiplications between powers of ten. This supports the view that positions and the shifts defined with respect to positions appeared conjointly—our oldest evidence coming from *The Nine Chapters on Mathematical Procedures*. Old-Babylonian sources, moreover, never attest to such a way of bringing the base into play—we return to this point later.

Once the multiplier has been multiplied by the order of magnitude of the first digit of the multiplicand (i.e. the digit of the highest degree), the next step consists in multiplying by the multiplier the very digit of the multiplicand corresponding to this order of magnitude and placing the result in the ‘middle position’. The next sentence of the text reads as follows:

When, with [the corresponding digit of the number] in the upper [row], one multiplies in these positions [a digit of the number] in the lower [row], we arrange the number obtained in the middle position. If we say ten (in the clause of the multiplication table used, see the literal translation of the table in figure 1), then, [placing the tens,] we go beyond [the position marked by the multiplied digit to the left]; what does not fill [ten] remains [in the position] identical to [the digit of the lower row multiplied]. 以上命下，所得之數列于中位。言十即過，不滿自如。”

**Figure 4.**
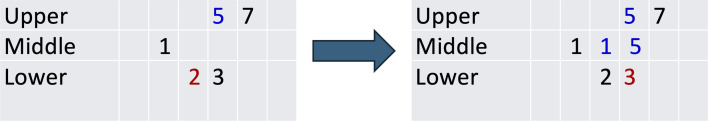
Multiplying in turn the two digits of the multiplier shifted (2 and then 3) by the first digit of the multiplicand, 5, and adding the intermediary results to the middle row, according to *Mathematical Canon by Master Sun.*

Here too, the graphical description of the operation highlights that the underlying numeration system is place-valued. If we consider our example in figure 4, when multiplying 5 by 2, the result is ‘ten’. Hence one places ‘1’ in the ‘middle position’, not above the 2, but ‘going beyond’ to the left. Similarly, when one multiplies 5 by 3, the result is fifteen (literally, in Chinese, ‘ten-five’). The ‘1’ is thus placed not immediately above the 3, but above to its left, whereas the ‘5’ is placed just above’ the ‘3’. Placing immediately above or to the left (‘beyond’) presupposes that the columns on the calculating surface have the numerical values of powers of ten—the power of the base. Conversely, this is how, in order to proceed, the computation relies on the base written as position.

**Figure 5.**
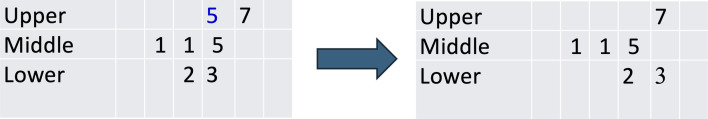
Deleting the digit of the multiplicand dealt with and shifting the multiplier backwards, to iterate the same steps as with the previous digit, according to *Mathematical Canon by Master Sun.*

Finally, echoing the place-value nature of the numeration system, the text of the algorithm formulates the principle of the iteration of these steps (figure 5):

Those of the positions (i.e., digits) in the upper [position] for which the multiplication is completed are first deleted. Those of the positions (i.e., digits) in the lower [position] for which the multiplication [by the related digit above] is completed are then all moved backwards [by one position]. 上位乗訖者先去之。下位乗訖者則俱退之。^10^

[The digits in] the upper and lower [rows] multiply each other until [the digits in the upper row] are exhausted, then [the operation] is over. 上下相乗，至盡則已。

To recapitulate what we have seen, the execution relies on the graphical properties of the sequence of digits that the notation of the base as position and the ensuing use of a place-value principle communicate to the representation of a number in this type of place-value numeration system. More broadly, however, the execution puts into play the tabular layout to which the inscription of the operands and of the successive intermediary results gives rise. The key elementary operation that is used turns the multiplication or the division of a number by the base or one of its powers into a horizontal shift of the representation of this number leftwards or rightwards against a backdrop in which columns have the meaning of powers of the base through the inscription of the numbers upon which one operates. The important observation is that the same algorithm could be used for any numerical value of the base, the only change being the extension of the multiplication table used. The algorithm, moreover, iterates exactly the same operations along the sequences of digits that represent the operands. The same properties hold for the algorithms given in this context to execute all the multiplicative operations, namely, for divisions and for root-extraction. As we shall now see, the algorithms attested to for the execution of multiplicative operations in Old-Babylonian sources rely on a similar sequence of digits in a completely different way.

## 3. Executing division-type operations according to Old-Babylonian sources

Let us return to the sexagesimal place-value notation that scribes learnt to use in scribal schools such as that of Nippur at the beginning of the second millennium BCE [1]. Each digit of this numeration system was written on clay tablets using two signs: one for the tens—to which I will refer using the letter ‘a’— and one for the units—to which I will refer using the letter ‘b’. The repetition of these signs indicated how many tens and how many units, respectively, the given digit contains. For example, ‘aaabb’ meant ‘32’. In this case, as in the case discussed above, the digits are written with signs that confer an inner structuring to the digit written. I will use both notations—the notation with letters and its meaning with Arabic numerals—to avoid giving the reader the impression that the digits of the numeration system were written using a decimal place-value notation.^11^ A sequence of digits was read through associating each digit to a power of 60 that was one unit higher than the digit to its right. I will separate such digits by a point, and hence ‘aaabb.abbbb’ refers to ‘32 times 60 plus 14’.

The way in which multiplications were executed with such a sexagesimal place-value notation is unclear, because we have only multiplication tables.^12^ One point is clear, nonetheless: these multiplication tables illustrate the floating-point character of the notation (see endnote ^1^ ). Indeed, they give the result of the multiplication of ‘bb’ (‘2’) by ‘aaa’ (‘30’), or of ‘bbbbbbb.aaa’ (‘7.30’) by ‘bbbbbbbb’ (‘8’), as being equal to ‘b’ (‘1’), and not as ‘b.0’ or ‘b.0.0’ (see respectively, [1, pp. 317, 319]).

Scribes trained in a school such as that of Nippur learnt to execute what for us is a division and did so by multiplying the dividend by the inverse of the divisor—to which present-day publications usually refer as its ‘reciprocal’ [23,24]. The practice shows that the reciprocal of a number was the number that multiplied by the original number gave ‘b’ (‘1’) as a result. Likewise, tables of reciprocals were part of those learnt by heart, and they gave, e.g. the reciprocal of ‘bb’ (‘2’) to be ‘aaa’ (‘30’), and that of ‘bbbbbbb.aaa’ (‘7.30’) as being equal to ‘bbbbbbbb’ (‘8’).^13^ In order to divide a number written in a sexagesimal place-value notation by, e.g. ‘aaa’ (‘30’), practitioners would therefore multiply the number by ‘bb’ (‘2’).

The computation of reciprocals is the subject of several tablets, probably because tables of reciprocals did not contain all the numbers by which one might have to divide; they would have proved useful moreover to anyone thinking about the algorithm for obtaining reciprocals. Such tablets are of two types: numerical tablets (such as CBS 1215, which only bears numbers) and tablets bearing mathematical problems (such as VAT 6505), both dating from the Old-Babylonian period and both unprovenanced, because found in the context of illegal excavations. These sources are essential for my purpose, since they highlight the features of the sequence of digits writing a number that were essential to compute a reciprocal and that are strikingly different from those that were put into play in the algorithms discussed in the previous section.

To explain this point, I will refer to tablet CBS 1215, which contains twenty-one computations of this kind.^14^ To compute the reciprocal of ‘bb.abbb.aa’ (‘2.13.20’), two key steps were used.

First, the scribe relied on the terminal digits of the sexagesimal place-value notation to identify factors of the number that would have reciprocals. In the case of our example, ‘bbb.aa’ (‘3.20’) is a number that occurs in the ordinary table for reciprocals, its reciprocal being ‘abbbbbbbb’ (‘18’) ([1, p. 316]). Moreover, ‘bbb.aa’ (‘3.20’) divides into ‘bb.abbb.aa’ (‘2.13.20’), since when a sexagesimal number ends with the sequence ‘bbb.aa’ (‘3.20’), it is divisible by the related number. Let us explain why:^15^ ‘bb.abbb.aa’ (‘2.13.20’) can be written as the sum of ‘bb.a.0’ (‘2.10.0’) and ‘bbb.aa’ (‘3.20’). The latter number is clearly divisible by ‘bbb.aa’ (‘3.20’). As for the former, a.0 (‘10.0,’ or in decimal notation, ‘600’) is divisible by ‘bbb.aa’ (‘3.20,’ or, in decimal notation, ‘200’), and any multiple of this as well. Any sequence of digits ending in b.0.0 (‘1.0.0,’ or in decimal notation, ‘3600’), moreover, is also divisible by ‘bbb.aa’ (‘3.20’, or, in decimal notation, ‘200’). The sum of all these numbers, ‘bb.abbb.aa’ (‘2.13.20’), is therefore divisible by ‘bbb.aa’ (‘3.20’). In other words, the terminal digits of a number written in sexagesimal place-value notation allowed practitioners to see which factors divided said number, and this was the key property of the notation that was used in the algorithm. For this reason, Friberg ([26:, p. 24]) calls this procedure the ‘trailing part algorithm.’

The second step consisted in then multiplying said number by the reciprocal of the factor identified. The result would be the original number in which the factor in question would have been eliminated; the practitioner could subsequently iterate the same sequence of two steps until reaching a number whose reciprocal was known. The result was finally obtained by multiplying the reciprocals of all the factors identified and eliminated one after the other.

In our example, once the factor ‘bbb.aa’ (‘3.20’) was identified, the number ‘bb.abbb.aa’ (‘2.13.20’) was multiplied by its reciprocal, ‘abbbbbbbb’ (‘18’), yielding ‘aaaa’ (‘40’). The table of reciprocals gave ‘b.aaa’ (‘1.30’) as the reciprocal of ‘aaaa’ (‘40’). As a result, the reciprocal of ‘bb.abbb.aa’ (‘2.13.20’) was obtained by multiplying ‘abbbbbbbb’ (‘18’) by ‘b.aaa’ (‘1.30’), which yielded ‘aabbbbbbb’ (‘27’).

We could apply a similar algorithm to a decimal place-value notation, which can be illustrated using the number 125. The sequence of digits ending in a 5, we know that the number is divisible by five. Multiplying 125 by 2 (the reciprocal of 5) yields 25 (in a floating-point notation). Twenty-five being again divisible by 5, one multiplies by its reciprocal 2, yielding 5 (again in a floating-point notation). The reciprocal of 125 would be obtained by multiplying 2 twice by itself, yielding 8.

The alternative algorithm that one finds in tablet VAT 6505 separates the tail of the place-value notation from the remaining part of the number ([23,24, p. 391]). For instance, the number ‘bb.abbb.aa’ (‘2.13.20’) is decomposed into two additive parts: ‘bb.a’ (‘2.10’) and ‘bbb.aa’ (‘3.20’). Each of them is then multiplied by the reciprocal of ‘bbb.aa’ (‘3.20’), yielding, respectively, ‘aaabbbbbbbbb’ (‘39’) and ‘b’ (‘1’), which are then added together to give ‘aaaa’ (‘40’). One may imagine that this algorithm made clearer the fact that the part of the sexagesimal place-value extension that remained, once the tail part had been taken away, was indeed divisible by the number represented by the tail part.

Whatever the case may be, we see that what matters in these two algorithms are rules of divisibility that are defined using only the terminal digits of the place-value notation of the number, as well as the determination of the factors that have a reciprocal. In base 60, the factors are those whose divisors are only 2, 3 and 5. In other words, what matters are the number-theoretical properties of the base 60, and the rules of divisibility that translate in terms of the number-theoretical properties of the terminal digits. The algorithms rely on the sexagesimal place-value expansions that represent numbers by an iterated operation that focuses on the terminal digits: the nature of the uniformity of the algorithm is different from what we have seen in the previous sections.

Interestingly, we also have tablets that testify to how other operations of the division type, i.e. square root and cube root extractions, were executed. They share exactly the same properties as the operations we have seen for the computation of reciprocals. Tablets YBC 6295^16^ and VAT 8547^17^ are both devoted to the computation of cube roots. The algorithms presented to execute this operation concentrate on the terminal digits of the representation of a number according to the sexagesimal place-value notation, looking for factors that are at the same time cubes and that have reciprocals. Multiplying the number whose root is sought by the reciprocal of the cube factor eliminates this factor, and then the same steps are carried out on the resulting number. In the end, the cube root is obtained as the cube root of all the factors successively eliminated. In fact, the algorithms presented in tablets YBC 6295 and VAT 8547 present exactly the same difference as those between CBS 1215 and VAT 6505.

Finally, Bruins [30] gives the translation of a tablet presumed to be from northern Mesopotamia ([24, p. 386]), which presents the computation of a square root.^18^ Again, the principles on which the algorithm relies are the same.

In conclusion, despite the fact that these operations of a division type are executed on a numeration system that is place-valued, the algorithms make use of the terminal digits of the sexagesimal expansions representing a number and exploit these digits with regard to number-theoretical properties related to the base 60. The way in which the base is put into play, in other words, differs strikingly from what we have seen above.

## 4. Conclusion

In this article, I have argued that ancient mathematical sources attest to the introduction of (at least) two distinct families of place-value numeration systems. In all the place-value numeration systems considered here, the base and its powers are represented using positions in a horizontal sequence of digits. The existence of a base is reflected by the repetitive character of the algorithms that draw on the related notations to execute operations—or multiplicative operations, to be more precise. The difference between these two families of numeration systems becomes clear when we consider the fact that their users ascribe a different role to the base number, as is manifest in the algorithms executing multiplicative operations on the basis of these notations. There are two distinct families of algorithms, indeed, in which computations rely on bases in completely different ways.

Some algorithms, like those associated with the Old-Babylonian sexagesimal place-value notations, exploit the number-theoretical properties of the value of the base, together with related features of the expansion of digits writing the number that the choice of the base makes prominent (like the terminal digits—usually referred to in publications as ‘the trailing part’—of its representation). By contrast, other algorithms rely only on the sequence of positions in which the operands are written. The latter could be applied to numbers written in numeration systems with *any* base (only the multiplication table would change), while the former are all the more appropriate to bases that have several divisors.

The computations’ iterative character is thus different in these two contexts. For instance, to compute a reciprocal, scribes in Old-Babylonian schools relied upon the terminal digits of the sexagesimal expansion of a number to identify regular divisors (which were not all merely digits) and multiply by their reciprocal; iterating this step rendered the result, factor by factor. At each step, what mattered was only the trailing part of the expansion. By contrast, divisions using decimal place-value notation in Chinese sources yielded quotients, digit by digit, through the iteration of the same steps of computation along the sequence of digits. With both types of use, the part played by the base, and hence its meaning, differed significantly.

These observations highlight the fact that the similarity between ancient Chinese and Sanskrit mathematical works with respect to the decimal place-value system is by no means dictated by the fact that there was only one way of operating on place-value notations. They further show that treating the Old-Babylonian sexagesimal place-value notation as similar to the decimal place-value system attested to by Chinese and Sanskrit sources overlooks that the meaning of the notation derives from its use. In this respect, the two types of place-value notation are quite different, the Old-Babylonian notation having disappeared at some point to be replaced by a sexagesimal place-value notation for which the execution of operations relied on the sequence of positions representing a number, rather than on the trailing part.

## Data Availability

All sources are made explicit.

## Footnotes

^1^
While it is impossible to give a complete bibliography on the subject, see Proust [1,2] as well as Robson [3, ch. 1]. Despite the fact that both authors insist on the floating-point character of the notation (see below for an explanation), their interpretative practice of this numeration system differs. For Proust, it is fundamentally a ‘floating-point notation’ (a term, to my knowledge, that was introduced by Donald Knuth—see [4, p. 671]), no underlying order of magnitude being attached to numerical signs, except when the scribe transformed a numerical sign written in sexagesimal place-value notation into the expression of a quantity. Accordingly, Proust translates the notation of a number by a mere sequence of digits. For Robson, by contrast, the scribe kept the order of magnitude in mind as he worked; in her translation of numerical signs, Robson represents this fact by inserting a semicolon after the digit she perceives to be that of the unit (see, for instance [3, p. 16]). Most Assyriologists have followed a similar practice in their translation, and Høyrup [5, p. 12] summarizes some of the systems used. While this issue is not essential for the questions addressed in the present article, I will follow Christine Proust in reproducing the numerical signs found in tablets as a mere sequence of digits. On the history of the interpretation of this numeration system, see Chaigneau [6].

^2^
While Arabic and Sanskrit sources also attest to the use of different types of sexagesimal place-value numeration systems, I will not consider them here, but will instead concentrate mainly on decimal place-value systems. My main point is to highlight that the fact of having a place-value notation is not enough to determine the part played by the base number. I thus need only consider two examples that show the differences in which I am interested.

^3^
This statement does not hold true for all sexagesimal place-value numeration systems. This is why I concentrate on Old-Babylonian sources.

^4^
Chemla (in dialogue with A. Keller and C. Proust) [12, pp. 62−67]. Ouyang & Proust [13] analyse cuneiform sources which indicate that, in some contexts, additive operations were carried out on a different numeration system.

^5^
Here and below, I rely on the critical edition given in Qian Baocong [14, vol. 2]. The passages quoted and translated below are on pp. 282−283. I will also mention the more recent edition [15]. Lam & Ang [16] give an English translation of the work. While the passages quoted below are translated in Lam & Ang [16, pp. 193−195], the translation given below is mine.

^6^
I follow the suggestion by Lam & Ang [16, p. 194, note 3], who consider that this sentence, which in the received editions features in the text for the multiplication algorithm translated below, was originally included here within the description of the numeration system.

^7^
In the illustrations I give below, I use Arabic numerals instead of rod-numerals. In fact, nowhere does the text suggest that the inner structuring of a digit in terms of rods was taken into consideration in the computation. In other words, if 9 is written with a horizontal rod for 5 and 4 vertical rods for the rest, the algorithms never make use of these specific rods to prescribe action with the 9. This is why there is no loss of information when one replaces rod-digits by Arabic digits. What is more, the text says nothing with respect to how the manipulations of digits during the execution took this inner structuring into account. This stands in contrast with later texts of algorithms with the abacus (see Chen [19]).

^8^
Here, and below, the Southern Song edition from 1213 has ‘upper position’上位, whereas the editions prepared at the end of the eighteenth century for the *Complete Library of the Four Treasuries* have ‘head/leading positions’ 頭位. I think the latter expression refers to the leading position of the number with rod-numerals in the position (upper or below) under consideration, in opposition to the expression of ‘upper position’, which contrasts with ‘middle/lower positions’. Like Qian Baocong [14, p. 283, note 2], I thus suggest keeping the reading ‘upper position’上位, which is what is specifically meant here. The difference is pointed out by Guo & Liu [15, p. 27, note 12], who nevertheless consider that both texts may conform to the original text.

^9^
See, for instance, Chemla [20].

^10^
Here the received editions have the sentence ‘六不積，五不隻.’ Following the suggestion by Lam & Ang [16, p. 194, note 3], I believe that this sentence was probably misplaced in the transmission and that it originally occurred earlier in the text (see endnote ^6^).

^11^
Around the year CE 1000, works like that by Kushyar ibn-Labban attest to the fact that the digits of sexagesimal place-value notations are written with a decimal place-value notation. Kushyar presents how to execute operations first on a decimal place-value notation, in which numbers are represented with a horizontal sequence of digits, and then on a sexagesimal place-value notation, in which numbers are represented with a vertical sequence of digits, each digit of the sexagesimal place-value notation being in turn written using a decimal place-value notation. Kushyar’s work testifies to a key change in the understanding of the sexagesimal place-value notation [21].

^12^
Proust [22] has pointed to mistakes in multiplication as suggesting that a computing instrument was used. However, these clues only indicate that numbers may have been dealt with in sequences of five digits at a time.

^13^
Proust [1, p. 316]. Høyrup [25] analyses an older (Sumerian) tablet that shows a division being executed using other systems of numeration. Here, I concentrate only on the sexagesimal place-value system.

^14^
I rely here on the interpretation of CBS 1215 given in Proust ([24, pp. 393−394], especially). Sachs [23] published the first analysis of this tablet. I follow Proust, however, in believing that he misinterpreted the algorithm underlying this tablet, probably because he deemed the algorithm to be the same one as was referred to in another tablet (VAT 6505).

^15^
Note that the explanation uses final digits 0, which are introduced only for the sake of clarity, since they are of no use in a floating-point system.

^16^
Neugebauer & Sachs [27, p. 42]. The text is quoted in Chemla [28, p. 21]. Chemla [28, pp. 2−6] sketches the opposition between this group of cuneiform texts and Chinese texts on division and root extractions.

^17^
Sachs [29, pp. 153−156].

^18^
The text is quoted in Chemla [28, p. 21].
